# Lipid‐ and glucose‐lowering effects of Rhamnan sulphate from *Monostroma nitidum* with altered gut microbiota in mice

**DOI:** 10.1002/fsn3.4100

**Published:** 2024-03-25

**Authors:** Yasuhito Shimada, Liqing Zang, Toshinari Ishimaru, Kaoru Nishiura, Koichi Matsuda, Ryota Uchida, Hiroko Nakayama, Izumi Matsuoka, Masahiro Terasawa, Norihiro Nishimura

**Affiliations:** ^1^ Department of Integrative Pharmacology Mie University Graduate School of Medicine Tsu Mie Japan; ^2^ Mie University Zebrafish Research Center Tsu Mie Japan; ^3^ Department of Bioinformatics Mie University Advanced Science Research Promotion Center Tsu Mie Japan; ^4^ Graduate School of Regional Innovation Studies Mie University Tsu Mie Japan; ^5^ Konan Chemical Manufacturing Co., Ltd. Yokkaichi Mie Japan

**Keywords:** constipation, diabetes, metabolic syndrome, microbiome

## Abstract

Rhamnan sulphate (RS) is a sulphated polysaccharide found in green algae such as *Monostroma nitidum* that exhibits various biological functions, including anticoagulant, antitumour, antiviral, and anti‐obesity properties. In our previous clinical trial, we demonstrated that RS intake improves constipation. However, no specific bacteria showed a significant (*p* < .05) change. Notably, these results were obtained after a short RS inoculation period of only 2 weeks. In the present study, to evaluate the long‐term effects of RS on the gut microbiota, we orally administered RS to BALB/c mice for 11 weeks, analyzed their blood biochemical data, and performed 16s rRNA‐sequencing. Oral administration of RS increased body weight with increased food intake, whereas plasma total cholesterol and fasting plasma glucose levels decreased. RS‐fed mice showed lower fasting insulin levels (*p* < .1) and decreased homeostatic model assessment for insulin resistance (HOMA‐IR, *p* < .0001), suggesting that RS improved insulin resistance. In the feces of mice, the amounts of acetic and propionic acids increased. In the gut microbiota, predictive metagenomic profiling using the phylogenetic investigation of communities by reconstruction of unobserved states (PICRUSt2) revealed functional alterations in Kyoto Encyclopaedia of Genes and Genomes (KEGG) pathways in RS‐fed mice. Corresponding to the blood glucose‐lowering effect, the glycolysis and tricarboxylic acid (TCA) cycle pathways were activated. In addition, the Firmicutes/Bacteroides (F/B) ratio, which may be associated with various health outcomes, was also reduced. These results suggest that the blood glucose‐lowering effect, improvement in insulin resistance, and lipid‐lowering effect of RS may be due to changes in the intestinal microbiota.

## Graphical abstract

Oral administration of Rhamnan sulphate (RS) led to a reduction in blood lipids and glucose levels. RS altered the gut microbiota and activated the glycolysis and TCA cycle pathways. RS reduced the Firmicutes/Bacteroidetes (F/B) ratio.

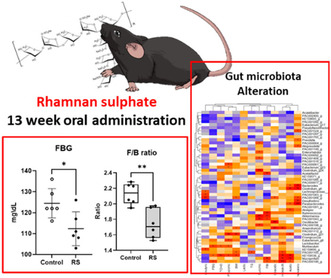

## 1 INTRODUCTION

Seaweeds have gained recognition as valuable sources of nutrients and bioactive compounds with numerous health benefits. They are rich in essential minerals, vitamins, dietary fibers, polyunsaturated fatty acids, and well‐known natural polysaccharides (Huang et al., 2022). Seaweed consumption has been associated with various positive effects on human health, including antioxidant and anti‐inflammatory properties, immune support, cardiovascular health support, anti‐obesity effects, and gut dysbiosis (Jiao et al., 2011; Lopez‐Santamarina et al., 2020; Yuan & Walsh, 2006). Various functional substances responsible for these effects have been identified in seaweeds, and their efficacy has been demonstrated through animal tests and human clinical trials. Polysaccharides have been extensively studied, providing valuable insights into their functional properties and potential applications in promoting human health. Fucoidan is well known for its anticancer functions (Turrini et al., 2023; Yamamoto et al., 1984) and has been used in adjuvant cancer therapy (Tsai et al., 2017). In addition, fucoidan and carrageenan have anti‐obesity properties, with a reduced Firmicutes/Bacteroidetes (F/B) ratio (Chin et al., 2019), a relevant marker of gut dysbiosis in obese populations, and an increase in specific gut bacteria (Shang et al., 2016, 2017).

*Monostroma nitidum*, commonly known as sea lettuce, is a green algal species belonging to the division Chlorophyta. *M. nitidum* is consumed in various cultures, especially in East Asian countries such as Japan and China. It is used in various dishes, including tempura, miso soup, and tsukudani (simmered in soy sauce and mirin). It has a mild flavor and high nutritional value and contains essential vitamins, minerals, and dietary fiber. Rhamnan sulphate (RS) was first identified in *M. nitidum* in 1998 (Harada & Maeda, 1998). In the following two decades, its potential health benefits and therapeutic properties gained attention, including anticoagulant (Li et al., 2012; Liu, Du, et al., 2018; Liu, Wang, et al., 2018), antiviral (Lee et al., 1999; Song et al., 2021; Terasawa et al., 2020; Wang et al., 2018), and anti‐inflammatory properties (Patil et al., 2022; Terasawa et al., 2022). In addition, it inhibits fatty liver and lowers lipid levels in an obese zebrafish model (Zang et al., 2015) and improves constipation by altering the gut microbiota in clinical trials (Shimada et al., 2021).

In our previous study, short‐term administration of RS for 2 weeks did not significantly change the human gut microbiota, with only partial functional changes detected through functional prediction using the phylogenetic investigation of communities by reconstruction of unobserved states (PICRUSt2) (Shimada et al., 2021). Seaweed‐derived compounds, including fucoidan (He et al., 2023; Zhang et al., 2022), can improve gut dysbiosis. Therefore, it was difficult to conclude that RS had minimal effects on the gut microbiota in our limited study. Therefore, in the present study, we conducted a relatively long‐term administration of RS in mice to analyze its impact on the gut microbiota. In addition, we have clarified the relationship between the lipid‐ and glucose‐lowering effects of RS, partially proved in our previous study (Shimada et al., 2021), and the alteration of gut microbiota caused by RS in the current study.

## 2 METHODS AND MATERIALS

### 2.1 Mouse experiment

RS (>95% purity) was prepared from *M. nitidum* (Terasawa et al., 2020) for this study. Five‐week‐old BALB/c mice were purchased from Japan SLC (Shizuoka, Japan) and housed under a 12‐h light/dark cycle at the Institute of Laboratory Animals at Mie University for 1 week. Six‐week‐old male mice were divided into two groups of six mice each, housed individually, and fed either a CE‐7 normal diet (ND; CLEA Japan, Tokyo, Japan) or CE‐7 supplemented with RS (250 mg/kg BW) for 11 weeks. During the feeding experiment, body weight and food intake were measured weekly. For blood chemistry analysis, the mice were fasted for 14 h before blood sampling.

All animal procedures were approved by the Ethics Committee of Mie University, performed according to the Japanese Animal Welfare Regulation Act on Welfare and Management of Animals (Ministry of Environment of Japan), and complied with international guidelines. Mice were euthanized with CO_2_ gas, the caecum was resected, and the contents were collected for 16s rRNA sequencing.

### 2.2 Blood chemistry

Blood glucose levels were measured using a handheld lab glucose glucometer (ForaCare Japan, Tokyo, Japan). Plasma triacylglyceride (TG) and total cholesterol (TCHO) were measured using Wako L‐type TG and TCHO kits (Fujifilm Wako Pure Chemical, Osaka, Japan). Insulin levels were determined using a mouse insulin enzyme‐linked immunosorbent assay (ELISA) kit (Mercodia AB, Uppsala, Sweden), according to the manufacturer's protocol. Homeostasis model assessment of insulin resistance (HOMA‐IR) was performed by dividing the product of fasting insulin (U/mL) and glucose (mmol/L) by 22.5.

### 2.3 Short‐chain fatty acid (SCFA) analysis in mouse stools

The concentrations of organic acids in the fecal contents were measured using high‐performance liquid chromatography (HPLC) with a UV‐4070 detector (JASCO, Tokyo, Japan), a guard column (KC‐G 6 B; Resonac), and two separation columns (Shodex Ionpack KC‐811; Resonac). Organic acid was separated at 60°C using the post‐column method (Park et al., 2017). For the mobile phase, 3 mM perchloric acid was used at a flow rate of 1.0 mL/min, and 125 mg/L BTB, a 5.3 g/L Na_2_HPO_3_ solution, was used for the post‐column reaction at a flow rate of 1.2 mL/min. Fecal samples were stored at −80°C until measurement. Fecal samples (100–120 mg) were suspended in 400 μL phosphate‐buffered saline (PBS) and homogenized using a homogenizer pestle. After centrifugation at 15,000 rpm at 4°C for 10 min, 200 μL of supernatant was recovered, and 200 μL of 5% perchloric acid was added to eliminate protein. The mixture was centrifuged at 15,000 rpm at 4°C for 10 min. Then, 200 μL of the deproteinized supernatant was recovered and added to 800 μL of the mobile phase. The supernatant mixed with the mobile phase was filtered by a membrane filter (0.45‐μm pore size). Then, 50 μL of the solution was used for HPLC analysis.

### 2.4 Genomic DNA extraction

Bacterial samples from the mouse caecum were freeze‐dried using a VD‐250R Freeze Dryer (Taitec, Saitama, Japan) and homogenized using a multi‐bead shocker (Yasui Kikai, Osaka, Japan). After homogenization, lysis solution F (Nippon Gene, Tokyo, Japan) was added, the mixture was heated at 65°C for 10 min, and the supernatant was collected after centrifugation (12,000 *g* for 2 min). Genomic DNA was isolated using a Lab‐Aid824s DNA Extraction Kit (Zeesan, Fujian, China), according to the manufacturer's protocol.

### 2.5 Sequencing of a 16s rRNA gene

Illumina MiSeq paired‐end sequencing of the hypervariable V3–V4 regions of 16S rRNA was performed at the Bioengineering Lab. Co., Ltd. (Kanagawa, Japan), as previously described (Shimada et al., 2021). Briefly, the DNA volume was determined using Synergy LX (Bio Tek, Winooski, VT, USA) and the QuantiFluor dsDNA System (Promega, Madison, WI, USA). A two‐step tailed pPCR approach was used according to the protocol for 16S metagenomic sequencing library preparation (Illumina, San Diego, CA, USA). The indexed libraries were analyzed with a fragment analyzer using the dsDNA 915 Reagent Kit (Advanced Analytical Technologies, Ames, IA, USA). The prepared libraries were used for paired‐end sequencing using MiSeq v3 reagents and 2 × 300‐bp reads on MiSeq (Illumina).

### 2.6 Analysis of bacterial composition in 16s rRNA datasets

Paired‐end reads of the 16S rRNA gene were assembled using QIIME2 (version 2022.8), as previously described (Shimada et al., 2021) using the DADA2 method (Bolyen et al., 2019; Callahan et al., 2016). Quality‐filtered reads were assigned to operational taxonomic units (OTUs) (100% identity) via de novo OTU picking and taxonomic assignment using the feature classifier plug‐in against the EzBioCloud 16S database (https://www.ezbiocloud.net/) (Yoon et al., 2017).

### 2.7 Microbiome analysis

α‐Diversity analysis using observed and Chao1 indices (Chao et al., 1993; Hughes et al., 2001) and β‐diversity analysis using unweighted Unifrac (Wong et al., 2016) were performed using QIIME2 with default parameters. PICRUSt2 version 2.3.0b (Langille et al., 2013) was used to predict functional gene products in the fecal microbiota based on the taxonomy obtained from the Kyoto Encyclopaedia of Genes and Genomes (KEGG) pathway database (Kanehisa et al., 2012; Okazaki et al., 2019). We calculated the Spearman correlation between the top 50 bacteria and phenotype changes using GraphPad Prism version 9.5.1 (GraphPad Software, San Diego, CA, USA) and visualized using the circlize (Gu et al., 2014) (version 0.4.15) and ComplexHeatmap (Gu et al., 2016; Gu & Hubschmann, 2022) (version 2.16.0) packages in R software, as previously reported (Metsalu & Vilo, 2015).

### 2.8 Statistical analysis

All results are presented as means with a standard deviation (SD). Data were analyzed via Student's *t*‐test using GraphPad Prism version 9.5.1 (GraphPad Software). Statistical significance was set at *p* < .05.

## 3 RESULTS

### 3.1 RS improved insulin resistance in mice

**FIGURE 1:**
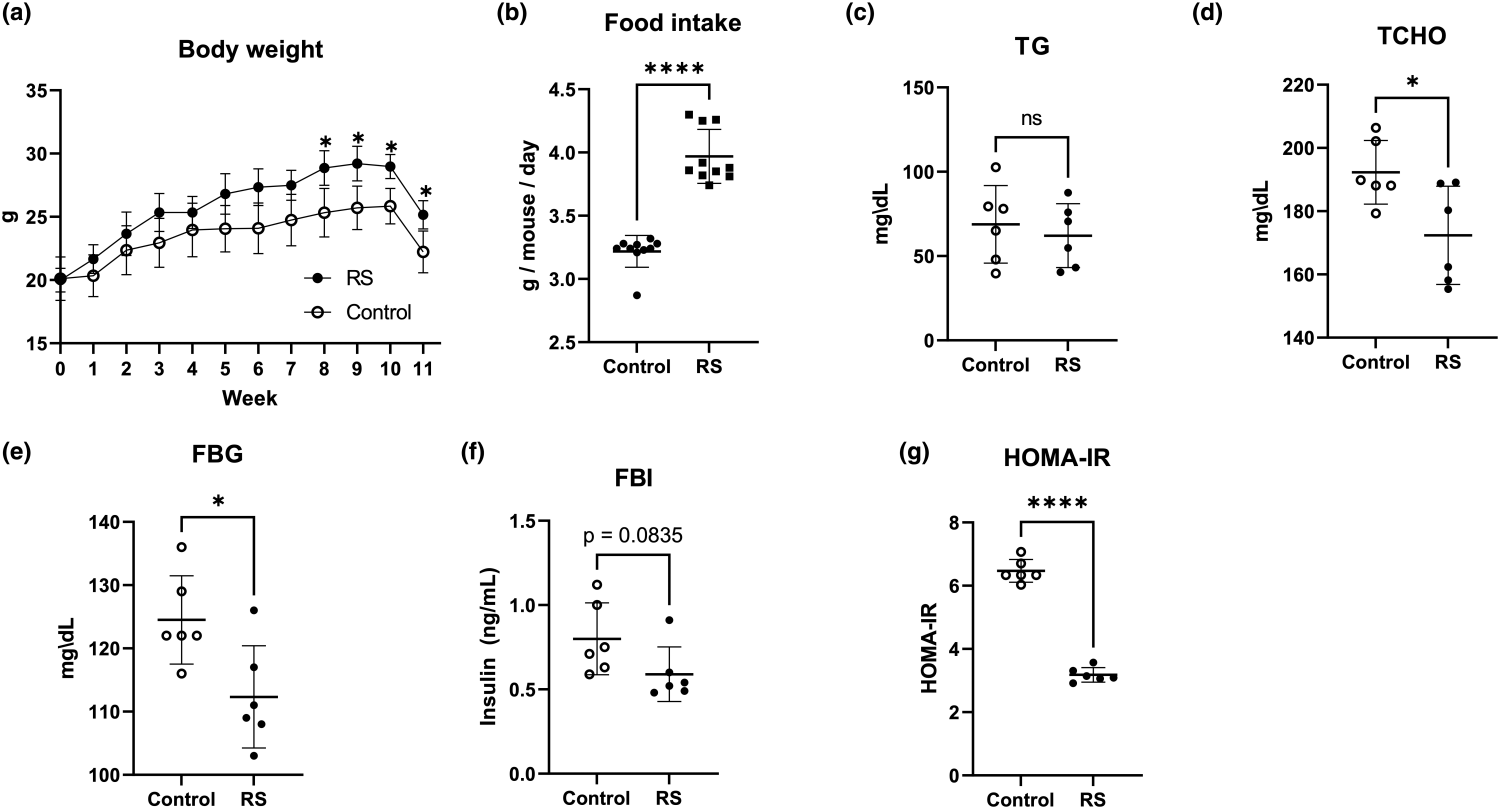
Oral administration of RS to mice. (a) RS administration increases body weight. *n* = 6; error bars indicate SD. (b) RS increases food intake in mice. Food intake was measured weekly throughout the feeding experiment. Each plot indicates the weekly average of six mice. *n* = 10; error bars indicate SD. (c, d). RS reduces plasma TCHO in mice. Plasma TG (c) and TCHO (d) were measured after the 11‐week feeding experiment. *n* = 6; error bars indicate SD. (e, f) Hypoglycaemic effects of RS. RS administration decreases FBG (e), FBI (f), and HOMA‐IR (g). *n* = 6; error bars indicate SD. *p < .05. ****p < .0001.

RS feeding significantly (*p* < .05) increased body weight compared to the control from week 8 (Figure 1a). After 11 weeks of experimental diet administration, the control group weighed 22.2 ± 1.7 g, whereas the RS group weighed 25.2 ± 1.1 g, representing weight gains of approximately 10% and 26%, respectively. On the measurement day of week 11, both groups experienced weight loss as they had fasted since the day before the blood biochemistry tests. As body weight increased, RS significantly (*p* < .0001) increased food intake (Figure 1b). Regarding plasma lipids, TG was not changed by RS feeding (Figure 1c), whereas TCHO was significantly (*p* < .05) reduced via RS (Figure 1d). Regarding glucose metabolism, RS significantly (*p* < .05) reduced the fasting blood glucose levels (FBG; Figure 1e). In addition, RS reduced fasting blood insulin (FBI) levels (Figure 1f) and significantly (*p* < .0001) lowered the HOMA‐IR index (Figure 1g), indicating that RS possessed hypoglycaemic properties with improved insulin resistance.

### 3.2 Microbiota alternation in RS‐fed mice

**FIGURE 2:**
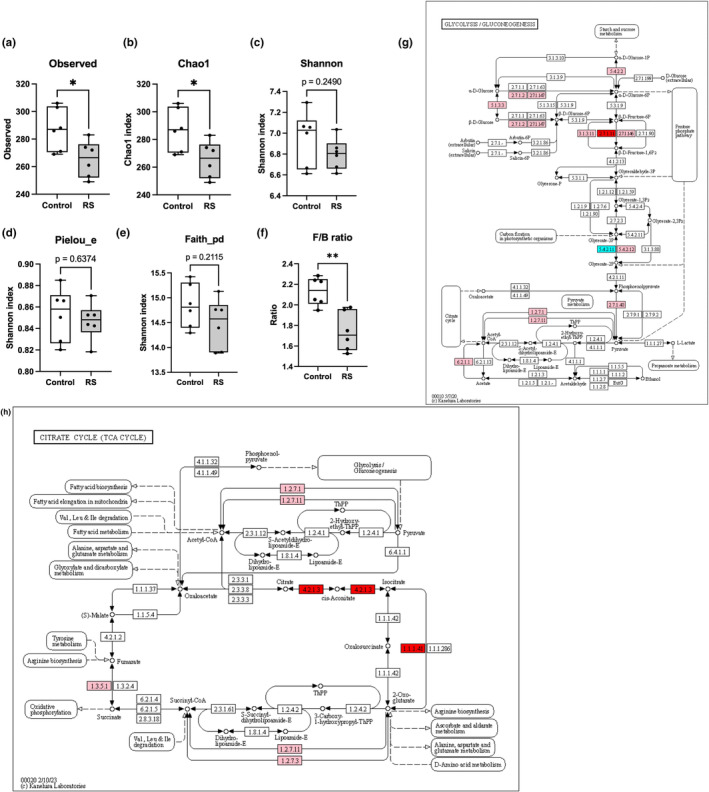
RS induced the alternation of gut microbiota. (a, b) RS reduces α‐diversity, shown using Observed (a) and Chao1 indices (b). (c) Shannon index (d), Pielou's evenness index (e), and Faith's phylogenetic α‐diversity. (f) RS reduces F/B ratio. *n* = 6; error bars indicate SD. (d, e) RS‐induced alteration of microbial KEGG metabolic pathways, glycolysis (g), and TCA cycle (h). Red and blue colors indicate upregulation and downregulation, respectively. KEGG pathway maps (ko00010 and ko00020) were adapted from http://www.kegg.jp/kegg/kegg1.html. *p < .05.

Based on the oral administration of fucoidan, a sulphated polysaccharide similar to RS, primarily affecting the caecal microbiota rather than the fecal microbiota (Wei et al., 2021), we performed 16s rRNA sequencing on the caecal microbiota of mice previously administered RS. The bacterial lists detected from the phylum level (level 2) to the family level (level 5) are presented in Tables [Link], [Link], [Link], [Link]. At the genus level (level 6), out of the 166 detectable bacteria, nine decreased (5.4%) and eight increased (4.8%) in number significantly (*p* < .05; Table S5) after RS feeding. At the species level (level 7), 403 bacteria were detected: 27 decreased (6.7%) and 22 increased (5.5%) in number significantly (*p* < .05; Table S6). Regarding bacterial diversity, RS significantly (*p* < .05) reduced α‐diversity in the observed and Chao1 indices (Figure 2a,b), while no significant differences were found in the Shannon index, Pielou's evenness index, or Faith's phylogenetic α‐diversity (Figure 2c–e). β‐Diversity using unweighted UniFrac is shown in Figure S1. In addition, the F/B ratio, a relevant marker of gut dysbiosis in obese populations (Chin et al., 2019), was significantly (*p* < .01) decreased by RS supplementation (Figure 2c).

### 3.3 Functional prediction from the altered gut microbiota

To visualize the effects of RS on the metabolic functions of the gut microbiota, we predicted bacterial metagenomes using PICRUSt2 (Douglas et al., 2020). The predicted proteins in each bacterium were classified into KEGG orthologue (KO) entities, identifying 4851 entities across all samples. Of these, 510 and 64 KOs were significantly (*p* < .05) upregulated and downregulated, respectively, in the RS group (Table S7). We then mapped the differentially expressed KOs to the KEGG Mapper to identify the altered pathways. In total, 199 pathways were identified, and the ratios between the number of KOs involving RS in each pathway and the total number of KOs in that pathway were calculated. Pathways with an occurrence rate of 5% were extracted (Table 1). The pathways involved in “Glycolysis/Gluconeogenesis (map00010)” and “Tricarboxylic acid (TCA cycle; map00020)” are represented in Figure 2d,e.

**TABLE 1:** KEGG metabolic pathways altered by RS administration.

Entry	Name	Number of hits	Total KO	Percentage of hits KO
map00633	Nitrotoluene degradation	7	22	31.8
map00020	Citrate cycle (TCA cycle)	15	67	22.4
map00430	Taurine and hypotaurine metabolism	5	29	17.2
map00730	Thiamine metabolism	6	36	16.7
map00230	Purine metabolism	19	117	16.2
map00520	Amino sugar and nucleotide sugar metabolism	17	106	16.0
map00250	Alanine, aspartate, and glutamate metabolism	11	70	15.7
map00540	Lipopolysaccharide biosynthesis	8	62	12.9
map00650	Butanoate metabolism	14	114	12.3
map01230	Biosynthesis of amino acids	29	238	12.2
map03070	Bacterial secretion system	9	74	12.2
map00620	Pyruvate metabolism	15	133	11.3
map00541	O‐Antigen nucleotide sugar biosynthesis	11	99	11.1
map01210	2‐Oxocarboxylic acid metabolism	9	82	11.0
map00010	Glycolysis/gluconeogenesis	17	156	10.9
map01200	Carbon metabolism	38	365	10.4
map05111	Biofilm formation – *Vibrio cholerae*	11	106	10.4
map00061	Fatty acid biosynthesis	4	39	10.3
map03060	Protein export	4	39	10.3
map00900	Terpenoid backbone biosynthesis	6	63	9.5
map00760	Nicotinate and nicotinamide metabolism	9	95	9.5
map00400	Phenylalanine, tyrosine, and tryptophan biosynthesis	7	74	9.5
map00330	Arginine and proline metabolism	10	107	9.3
map00640	Propanoate metabolism	9	97	9.3
map00920	Sulfur metabolism	10	109	9.2
map00030	Pentose phosphate pathway	8	88	9.1
map00720	Carbon fixation pathways in prokaryotes	19	211	9.0
map00910	Nitrogen metabolism	6	68	8.8
map00190	Oxidative phosphorylation	19	223	8.5
map00340	Histidine metabolism	4	47	8.5
map00130	Ubiquinone and other terpenoid‐quinone biosynthesis	5	59	8.5
map00220	Arginine biosynthesis	5	60	8.3
map00680	Methane metabolism	16	195	8.2
map01232	Nucleotide metabolism	10	123	8.1
map00240	Pyrimidine metabolism	9	111	8.1
map01250	Biosynthesis of nucleotide sugars	17	211	8.1
map00051	Fructose and mannose metabolism	9	112	8.0
map00052	Galactose metabolism	6	78	7.7
map03018	RNA degradation	6	79	7.6
map00740	Riboflavin metabolism	4	54	7.4
map02040	Flagellar assembly	4	55	7.3
map01240	Biosynthesis of cofactors	27	375	7.2
map00630	Glyoxylate and dicarboxylate metabolism	7	104	6.7
map02026	Biofilm formation – *Escherichia coli*	4	61	6.6
map00300	Lysine biosynthesis	3	48	6.3
map02010	ABC transporters	31	515	6.0
map00500	Starch and sucrose metabolism	6	106	5.7
map02020	Two‐component system	28	499	5.6
map00270	Cysteine and methionine metabolism	7	125	5.6
map00260	Glycine, serine, and threonine metabolism	6	109	5.5
map00360	Phenylalanine metabolism	4	74	5.4
map00564	Glycerophospholipid metabolism	6	115	5.2

### 3.4 Correlation of RS‐fed gut microbiota with body weight change, blood chemistry, and SCFA levels

**FIGURE 3:**
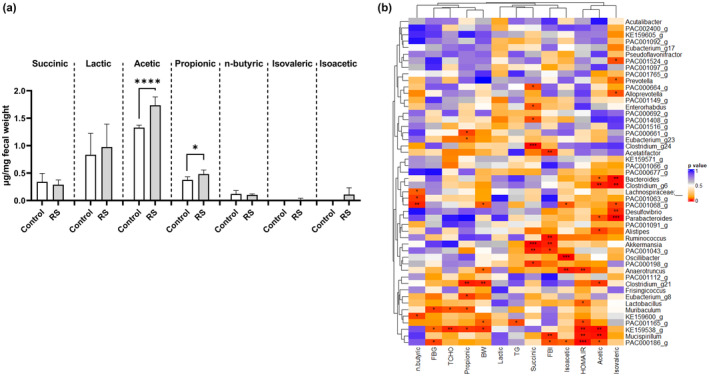
Relationships between blood chemistry, fecal SCFAs, and gut microbiota. (a) Measurement of fecal SCFAs. *n* = 6; error bars indicate SD. (b) Spearman analysis of the correlation of microbiota at the genus level using blood chemistry and fecal SCFAs. Red color indicates high correlation. *n* = 6, *p < .05, **p < .01, ***p < .001, ****p < .0001.

We measured the fecal levels of seven SCFAs in the control and RS‐fed mice. As shown in Figure 3a, acetic and propionic acid levels were significantly (*p* < .0001 and *p* < .05) increased by RS feeding. Next, the relationships of the top 50 most abundant genera in all samples with body weight change, plasma TG, plasma TCHO, FBG, FBI, HOMA‐IR, and fecal SCFA levels were analyzed using the Spearman correlation coefficient. In Figure 3b, the bacteria with red colors and asterisks are highly correlated (*p < .05, **p < .01, and ***p < .001) with each phenotype (blood chemistry and fecal SCFAs). The bacteria corresponding to the lower right of the heatmap showed a strong correlation with blood glucose control and the amount of fecal acetic acid.

## 4 DISCUSSION

### 4.1 Phenotypic changes by RS administration

Oral administration of RS to normal mice significantly (*p* < .05) increased body weight (Figure 1a), whereas plasma TCHO levels decreased (Figure 1d). This lipid‐lowering effect was consistent with previous studies using diet‐induced obese zebrafish (Zang et al., 2015) and ApoE mice (Patil et al., 2022). In addition, RS administration decreased the FBG, insulin, and HOMA‐R levels (Figure 2). A mild blood‐glucose‐lowering effect has also been observed in clinical trials (Shimada et al., 2021). The high‐molecular‐weight sulphated polysaccharide fiber RS may be utilized by specific gut microbiota when orally administered, increasing specific gut microbiota and exerting lipid‐ and glucose‐lowering effects. Regardless of its effect on body weight, RS, with its ability to lower both blood lipid and glucose levels, could also be a powerful functional food for promoting human health.

The weight gain effect of RS in the normal diet group, as observed in this study, is not seen in the NSY/HOS (Shimada et al., 2021) or ApoE^−/−^ strains (Terasawa et al., 2023), which is puzzling. Moreover, the BALB/c strain is one of the least likely to become obese with dietary load (Li et al., 2020), and yet RS increased their weight. Considering that appetite increased while blood glucose and plasma TCHO significantly decreased and plasma TG remained unchanged, this phenotype could possibly be growth rather than obesity. Regrettably, we did not measure height. Alternatively, it could be increased muscle mass. There are reports of seaweed and skeletal muscle hypertrophy (Korivi et al., 2019) and muscle‐strengthening effects via fucoidan (McBean et al., 2021), so RS may also have a muscle‐mass‐increasing effect.

The mechanisms underlying the lipid‐ and glucose‐lowering effects are likely multifaceted and diverse. Potential mechanisms include the previously reported promotion of oxidation in the liver via RS (Zang et al., 2015), increased fecal volume (due to absorption inhibition) in mice and humans (Shimada et al., 2021), and lipid lowering with anti‐inflammatory effects in ApoE mice (Patil et al., 2022). In addition, we identified that the effect of RS on the gut microbiota contributed to the blood glucose‐lowering effect. Due to its high molecular weight, RS is expected to inhibit intestinal sugar absorption. However, this alone insufficiently improves insulin resistance, as observed in this study. Dysbiosis‐induced inflammation exacerbates insulin resistance (Scheithauer et al., 2020), whereas SCFAs, metabolites produced by the gut microbiota during the fermentation of dietary fiber, can improve insulin sensitivity by enhancing glucose metabolism and insulin signaling (den Besten et al., 2013; Portincasa et al., 2022). Among SCFAs, acetate, which was increased in feces (Figure 3a), has beneficial effects on host energy and substrate metabolism. It reduces overall body fat deposition and decreases systemic levels of inflammatory cytokines while increasing energy expenditure and fatty acid oxidation (Hernández et al., 2019). The anti‐inflammatory effects of RS (Okamoto et al., 2019; Terasawa et al., 2022) and the increase in acetate revealed in this study may be mediated by the effect of RS on the gut microbiota, subsequently contributing to the blood glucose‐lowering effect. In fact, no studies have directly proven the relationship between fecal acetate and insulin resistance, although there have been reports of improvements in the gut microbiota and insulin resistance by administering acetate (Perry et al., 2016).

### 4.2 RS effects on the gut microbiota

Metabolic functional analysis of the altered gut microbiota revealed an activated glycolytic pathway (Table 1 and Figure 2d). This is consistent with the phenotypic changes observed in the improvement of insulin induced by RS. TCA cycle activation was also anticipated (Figure 2e), suggesting lipid beta‐oxidation activation in the gut environment, which may contribute to the decrease in lipid levels caused by RS. In terms of the effect of RS on individual bacteria, the order Deferribacterales increased significantly (*p* < .01) in response to RS (Figure S2). Although the involvement of Deferribacterales in the improvement of insulin resistance or obesity has not been reported, an increase in Deferribacterales occurs in the guts of mice and rats fed a high‐fat diet (Hamilton et al., 2015; Jiao et al., 2018; Liu et al., 2019). These results may seem to contradict our own at first glance. However, it is possible that Deferribacteriales are proliferating in response to weight gain induced via RS or that they are increasing to inhibit intestinal lipid absorption during high‐fat diet loading, which could, in turn, reduce plasma TCHO in a normal diet, as in this study. Further research is needed to clarify these mechanisms.

The results of the Spearman correlation analysis suggested the involvement of multiple bacteria related to the improvement in insulin resistance following RS administration (Figure 3b). This analysis showed a high correlation between “faecal acetic acid and Clostridium_g6” and “FBI and Akkermansia”. These associations have been demonstrated previously (Dao et al., 2016; Everard et al., 2013; Louis & Flint, 2009; Zhang et al., 2018), making this analysis convincing. Furthermore, in the lower right part of the heatmap, bacteria such as KE159538_g, Mucispirillum, and PAC000186_g, whose functions are unknown, strongly correlate with increased FBI, HOMA‐IR, and acetic acid in the feces. KE159538_g belongs to the Lachnospiraceae family, Mucispirillum to the Deferribacteraceae family, and PAC000186_g to the Muribaculaceae family. Except for Deferribacteraceae, there were associations in the literature between these families and acetic acid production or insulin resistance. For example, when cooked pea seed coats are administered to HFD‐fed mice, they improve glucose intolerance, increase serum acetate levels, and increase Lachnospiraceae in the gut (Hashemi et al., 2017). In addition, when sciadonic acid, which has a remedial effect on type 2 diabetes mellitus, is administered to mice, Muribaculaceae increases, along with increased fecal acetic acid (Chen et al., 2022). Although the direct mechanism between these bacteria, insulin resistance, and fecal acetic acid is not yet known, our findings suggest the existence of a mechanism common to RS.

RS significantly (*p* < .05) reduced the diversity of the gut microbiota, as evidenced by both the Observed (Figure 2a) and Chao1 (Figure 2b) indices. Generally, a decrease in diversity suggests the possibility of certain bacteria increasing, whereas the number of other bacteria decreases, indicating a disruption of gut balance and an increased health risk. Gut bacteria play a crucial role in food digestion and nutrient absorption. Therefore, a decrease in diversity may hinder the metabolism of specific nutrients and potentially reduce the absorption of nutrients, such as carbohydrates. In fact, many dietary manipulations and supplementations show positive effects in model animals (Cantu‐Jungles & Hamaker, 2023) and in clinical trials (Su et al., 2022; Zhang et al., 2015) while also reducing the α‐diversity. Among these, dietary fiber supplementation, similar to RS, increases specific gut bacteria and reduces α‐diversity, yet it also demonstrates glucose‐lowering effects (Zhao et al., 2018). In contrast, the significant (*p* < .01) decrease in the F/B ratio (Figure 2c), an indicator of the gut microbiota associated with obesity, suggests an anti‐obesity effect of RS through modulating the gut microbiota (which may be masked by increased food intake in this study).

In humans, changes in the gut microbiota induced by diet can occur within a short period of time (5 days), and calorie intake changes correspondingly (David et al., 2014). In our current study, similar changes may occur shortly after oral administration of RS. However, in our previous four‐week administration trial of RS in mice, there were no significant (*p* < .05) changes in blood lipids or glucose levels (Shimada et al., 2021), so we conducted an 11‐week administration trial in this study. It is highly likely that there is a time lag between changes in the gut microbiota and phenotypic changes in these mice. Indeed, in the analysis of fucoidan effects on the gut microbiota in HFD mice, administration was conducted for 5–8 weeks (Chen et al., 2019; Huang et al., 2021).

## 5 CONCLUSION

After administering RS to mice over a relatively extended period, we observed improvements in insulin resistance, along with decreased fasting blood glucose and plasma TCHO levels. Functional analysis of the gut microbiota revealed the corresponding activation of both the glycolysis and TCA cycle pathways. This suggests that the effects of RS may be mediated by changes in the intestinal microbiota.

## AUTHOR CONTRIBUTIONS

**Yasuhito Shimada:** Conceptualization (equal); data curation (lead); formal analysis (lead); investigation (lead); methodology (lead); project administration (supporting); supervision (equal); visualization (supporting); writing – original draft (lead). **Liqing Zang:** Formal analysis (supporting); investigation (supporting); methodology (supporting); writing – review and editing (equal). **Toshinari Ishimaru:** Formal analysis (supporting); visualization (lead). **Kaoru Nishiura:** Data curation (supporting); formal analysis (supporting); methodology (supporting). **Koichi Matsuda:** Resources (equal). **Ryota Uchida:** Resources (equal). **HIroko Nakayama:** Methodology (supporting). **Izumi Matsuoka:** Methodology (supporting). **Masahiro Terasawa:** Data curation (supporting); formal analysis (supporting); investigation (supporting); project administration (supporting); writing – review and editing (equal). **Norihiro Nishimura:** Conceptualization (equal); project administration (lead); supervision (equal).

## CONFLICT OF INTEREST STATEMENT

The authors declare that they do not have any conflicts of interest.

## ETHICS STATEMENT

This study was approved by the Institutional Review Board of Mie University.

## Supporting information


Figure S1

Table S1

Table S2

Table S3

Table S4

Table S5

Table S6

Table S7

## Data Availability

The data that support the findings of this study are available on request from the corresponding author.
