# Supplementary material for: Lipid‐ and glucose‐lowering effects of Rhamnan sulphate from Monostroma nitidum with altered gut microbiota in mice
Source: Food Sci Nutr. 2024 Mar 25;12(6):4342–52. doi: 10.1002/fsn3.4100 (PMC11167150; doi:10.1002/fsn3.4100)
Supplement: Supplementary file 1 — Figure S1 [file FSN3-12-4342-s004.docx]

**FigureS1.**


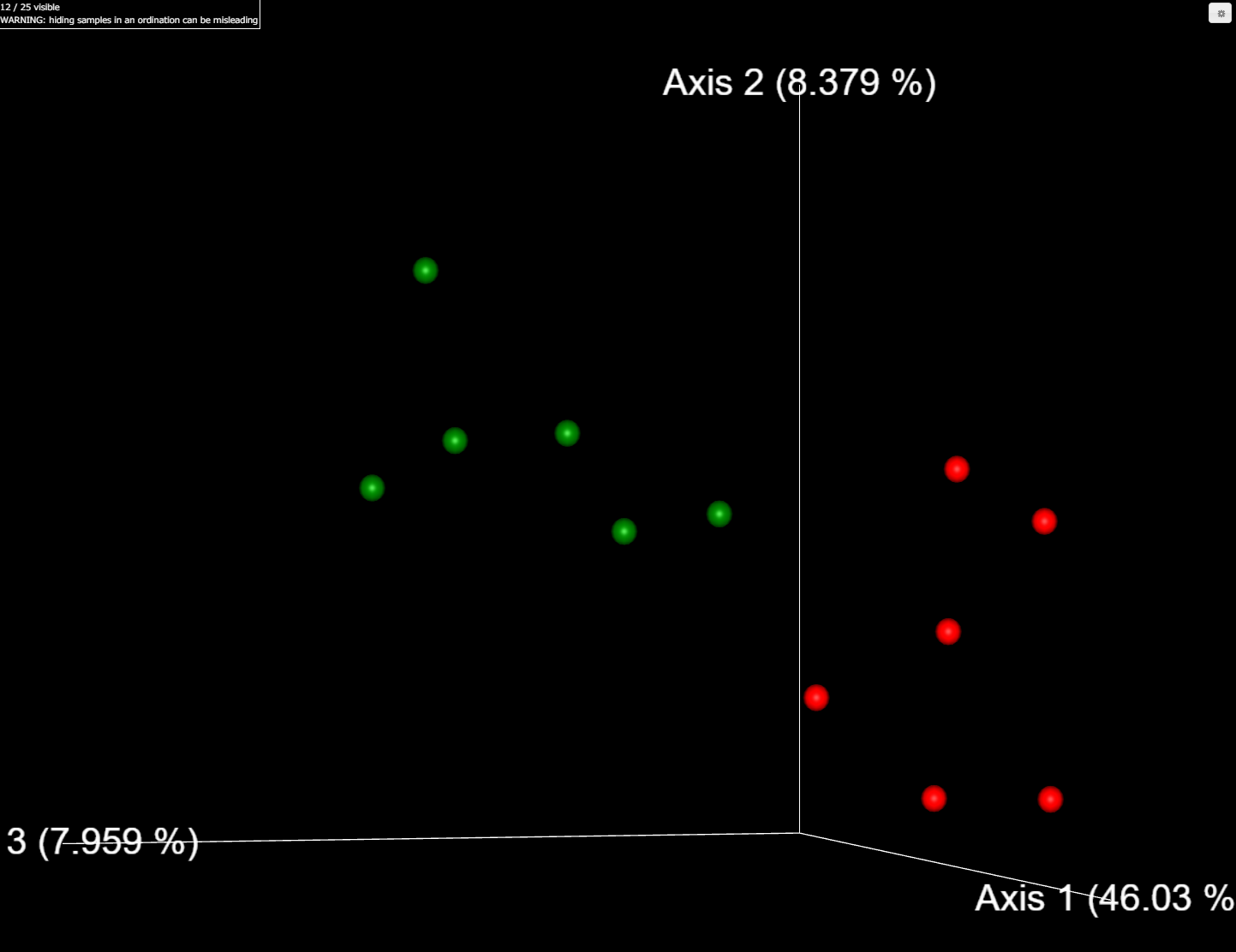


**FigureS1. Beta diversity using unweighted Unifrac.** Green and red indicate control and RS, respectively.

**Figure S2.**

**FigureS2. RS increases Defferibacterales order.** Defferibacterales is increased in RS group. ***p* < 0.01, n = 6.
